# Neuropsychodynamic Approach to Depression: Integrating Resting State Dysfunctions of the Brain and Disturbed Self-Related Processes

**DOI:** 10.3389/fnhum.2018.00247

**Published:** 2018-06-27

**Authors:** Heinz Boeker, Rainer Kraehenmann

**Affiliations:** ^1^Department of Psychiatry, Psychotherapy and Psychosomatics, Psychiatric Hospital, University of Zürich, Zürich, Switzerland; ^2^Center for Psychiatry, Psychotherapy, and Psychoanalysis, Psychiatric University Hospital Zurich, Zürich, Switzerland

**Keywords:** depression, neuropsychodynamic psychiatry, self, resting state, default mode network (DMN), mechanism-based approach, psychotherapy

## Abstract

A mechanism-based approach was developed focusing on the psychodynamic, psychological and neuronal mechanisms in healthy and depressed persons. In this integrative concept of depression, the self is a core dimension in depression. It is attributed to negative emotions (e.g., failure, guilt). The *increased inward focus* in depression is connected with a *decreased environmental focus*. The development of neuropsychodynamic hypotheses of the altered self-reference is based on the investigation of the emotional-cognitive interaction in depressed patients. It may be hypothesized that the increased negative self-attributions—as typical characteristics of an increased self-focus in depression—may result from altered neuronal activity in subcortical-cortical midline structures in the brain, especially from hyperactivity in the cortical-subcortical midline regions and hypoactivity in the lateral regions. The increased resting state activity in depression is especially associated with an increased resting state activity in the default mode network (DMN) and a dysbalance between DMN and executive network (EN) activity. Possible therapeutic consequences of the neuropsychodynamic approach to depression involve the necessary emotional attunement in psychotherapy of depressed patients and the adequate timing of therapeutic interventions. The hypotheses which have been developed in the context of the neuropsychodynamic model of depression may be used for more specific psychotherapeutic interventions, aiming at specific mechanisms of compensation and defence, which are related to the increased resting state activity and the disturbed resting state-stimulus-interaction.

## Introduction

The development of a neuropsychodynamic theory of depression should take into account the correlation between neuroscience and psychoanalysis, the complexity of subjective experience in the first-person perspective, and the impossibility to localize this subjective experience in specific brain regions. The starting point for the neuropsychodynamic theory of depression is a psychic-orientated term of localization, which considers the biological, psychological and social dimension of depression, with the differentiation between higher and lower regions of functions being of only secondary importance. In this neuropsychodynamic concept, the different modes of correlation between biological, psychological and social dimensions are central. Therefore, contrary to a merely biologically-oriented perspective in which the brain is generally looked upon as isolated from the psychosocial context, the psychodynamic perspective looks upon the brain as “embedded” in the psychosocial context (Northoff, 2000, 2004, 2013; Northoff and Boeker, 2006; Northoff et al., 2004). In this view, first-person neuroscience should indeed search for non-localistic ways of finding correlations between subjective experience and neuronal conditions. Therefore, neuronal integration over different brain regions, rather than neuronal localization in one or more regions, will be discussed in the following neuropsychodynamic framework of depression.

## Psychodynamic Key Features of Depression

In view of the development of the variety of psychoanalytic theories of depression (overview in Brenner, 1991; Mentzos, 1995, 2009; Böker, 1999, 2005; Gabbard, 2005, 2014; see also Force, 2008; for an operationalized research approach to Psychodynamic Psychotherapy of depression), we will focus on the essential dimensions of depression, which are related to Freud’s outstanding contribution “Mourning and Melancholia” (Freud, 1917). Contrary to mourning, in which the loss of the significant other is elevated by symbol formation (Segal, 1957), the depressive syndrome does not have a symbolizing function, but is the consequence of the inhibition or the somatopsychic-psychosomatic dead-end of depression (Gut, 1989; Böker, 2002, 2003a). At an early stage, Freud differentiated depression, paraphrased as “melancholia,” from mourning (Trauerarbeit): “In mourning it is the world which has become poor and empty; in melancholia it is the ego itself.” (Freud, 1917, p. 246). This citation underlines the functional perspective on depressive symptoms: a primary useful reaction to loss (withdrawal of the mourning person to stabilize one’s self) develops into becoming dysfunctional in the further course (lack of emotional resonance, predominance of negative, dysfunctional cognitions and extreme withdrawal behavior). Freud stressed the ego-regression from object cathexis to narcissism and pointed to the loss of reality function connected with this:

“*‥.; then, owing to a real slight or disappointment coming from this loved person, the object-relationship was shattered. The result was not the normal one of a withdrawal of the libido from this object and a displacement of it on to a new one, but something different, for whose coming-about various conditions seem to be necessary. The object-cathexis proved to have little power of resistance and was brought to an end. But the free libido was not displaced on to another object; it was withdrawn into the ego. There, however, it was not employed in any unspecified way, but served to establish an identification of the ego with the abandoned object. Thus, the shadow of the object fell upon the ego, and the latter could henceforth be judged by a special agency, as though it were an object, the forsaken object. In this way an object-loss was transformed into an ego-loss and the conflict between the ego and the loved person into a cleavage between the critical activity and the ego as altered by identification*”

*—(Freud, 1917, p. 249)*.

Freud’s original phenomenological distinction between mourning and depression is supported by recent neurobiological studies. For example, Gündel et al. (2003) showed that mourning is mediated by a distributed brain network related to affect processing, mentalizing, emotional memory, face processing, visual imagery and autonomic regulation. Other studies (O’Connor et al., 2002, 2008; Najib et al., 2004) also found neurobiological changes in mourning that are distinct from changes in depression (e.g., activation of nucleus accumbens, activation of amygdala, variation in the monoamine oxidase A (MAO-A) promoter gene, variation in heart rate variability).

The important relation between loss of the object and loss of the self will be focussed on in a neuro-scientific perspective (see Boeker and Northoff, 2018). Along this line, we will refer to the following “psychodynamic essentials of depression”:

1. Reactivation of earlier experiences of loss in childhood.
2. Introjection of the lost objects in childhood in connection with negative emotions.
3. Loss of object relations in connection with increased self-focussed attention.

Studies and theories which are influenced by object-relations psychoanalysis and infant research are of outstanding importance for the neuropsychodynamic approach of depression described in this article. In fact, previous studies consistently showed that mental representations of care-giving relationships are key components in understanding the development of a vulnerability to depression (Stolorov, 1986; Adroer, 1998; Malancharuvil, 2004; Fonagy et al., 2007). Most importantly, the *early loss of objects in childhood* is part of the bio-psycho-social vulnerability of persons likely to develop a depressive disorder in later life (Jacobson, 1971; Kernberg, 1976; Bowlby, 1980; Lichtenberg, 1983; Stern, 1983, 1985; Gut, 1989; Blatt, 1998). The fixation on the mental representation of the lost objects involves great psycho-energetic exertion, which Freud compared to an “open wound” taking psychic energy from the self and object representations. This regressive process leads to both the outer world and the self being completely depleted of representations. Besides the early loss of objects in childhood and the increased inner focus, the loss of actual object relations represents the third psychodynamic key feature of depression. The mental representation of the lost object includes a disregarding encountering of other objects in the actual environment of the adult. Others are exclusively or predominantly perceived in the perspective of the lost object. Depressed persons develop an increased inner focus and are no longer able to focus on the outer world. This *self-focussed attention* is the focus of perception of interpersonal relationships: the self-focused attention in depressed patients (Ingram, 1990) is often connected with interactional vicious circles which may cause an increasing helplessness of both partners. Previous studies on social interaction (Coyne, 1976a,b, 1985; Coyne et al., 1987; Boeker, 2004), for instance, showed that the environmental response may play an important role in the maintenance of depressed behavior; further, that partners are important for the self identity of the depressive patient in a much stronger way than for non-depressed controls; and finally, that half of the depressive patients “construed” the partner as similar to the self and similar to the ideal self. With regard to the sensory perception, it can be assumed that the attention shifts from the exteroceptive sensory system (signalizes environmental events and the relationship between the subject and her/his environment) to the interoceptive system, which processes the stimuli of the body.

A great number of studies with different measures and methodologies underline the self focused perception in depression. The results converge in an increased and possibly altered level of self focused attention in depression (Ingram, 1990). It is unclear up to now, whether this increased self focus exclusively exists on an explicit, conscious level—according to our definition—or already on an implicit, unconscious level.

The self in depression is attributed to negative emotions (failure, guilt, hypochondriacal fear of illness and death, in some cases connected with depressive delusion). Positive emotions are no longer connected with ones own self. These scrupulous tendencies correlate with the number of former suicide attempts and are risk factors for suicidal behavior.

The cognitive processing of the own self (rumination) increases the depressive mood and develops into an increasingly dysfunctional mechanism of compensation which is connected with the self-focussed attention and a repetitive focus of ones own negative emotions (Ingram, 1990; Treynor et al., 2003; Rimes and Watkins, 2005). It may be hypothesized that the increased inward focus in depression corresponds with psychodynamic processes, especially introjections and identification of the self with a lost object. The connection between introject and self with negative affects which is postulated in a psychodynamic perspective corresponds to the phenomenological/psychopathological description of the association of the self with negative emotions. Finally, the significance of the actual loss of objects corresponds with the increased cognitive processing of the self because the cognitive processing no longer focuses on the actual loss, but on the loss of objects with which the self is identified (“decreased environmental focus”).

## Neuropsychodynamic Concept of Psychodynamic Defence Mechanisms in Depression

Neuronal integration characterizes the coordination and modulation of neuronal activity over multiple brain regions. The interaction between brain regions far apart from each other is considered necessary for the development of specific functions such as emotions or cognition (see Price and Friston, 2002; Friston, 2003). It may be hypothesized that defence mechanisms are complex emotional-cognitive interactions that are not localized in specific or isolated brain regions but depend on the interaction between different brain regions, i.e., neuronal integration, or connectivity.

There is a distinction between functional and effective connectivity (see Friston and Price, 2001, p. 277): functional connectivity represents the “correlation between distant neurophysiological events” which may depend either on direct interaction between these events or on different factors which modulate different events. In the first case, the correlation results from the interaction itself; in the second case, the correlation may result by means of different factors, e.g., stimulus-related procedures with common input or stimulus-induced oscillations. In contrast to functional connectivity, effective connectivity describes the directed interaction between brain regions. Effective connectivity relates to the directed influence of one neuronal system to another, either at the synaptic level or at the level of neuronal interaction in different regions (macro-level, see Friston and Price, 2001). In the further course of this article, connectivity at the macro-level is assumed.

On the basis of connectivity, the neuronal activity between brain regions far away from each other can be adapted, coordinated and harmonized. This coordination and adaptation relies on certain principles of neuronal integration (Northoff, 2004; Northoff and Boeker, 2006). These principles represent functional mechanisms which play an important role in the organization and coordination of neuronal activity in different brain regions.

Four of these principles have been investigated in our neuroimaging studies on emotional-cognitive interaction:

1. Top-down/bottom-up modulation and somatization.
2. Reciprocal modulation and introjection.
3. Modulation through functional unit and sensori-motor regression.
4. Self-referential processing, ego-inhibition, and decoupling of the self.

It may be hypothesized that each of these four principles of neuronal integration may be associated with specific mechanisms of defence and compensation.

### Top-Down/Bottom-Up Modulation and Somatization

Top-down modulation can be described as modulation of hierarchically lower-level brain regions by higher-level regions. Thus, top-down modulation resembles the concept of “re-entrant circularity” (Tononi and Edelman, 2000) and feedback-modulation (Lamme, 2001). These concepts focus on exchange of information and adaptation of neuronal activity in a certain brain region in accordance with another remote region. As a result, neuronal activity in a lower region can be adapted, filtered and attuned in accordance with neuronal activity in the higher region. Typical examples of top-down modulation are: modulation of neuronal activity in subcortical regions by cortical regions; modulation of subcortical basal ganglia (e.g., nucleus caudatus and striatum) by premotor/motoric cortical regions (see Masterman and Cummings, 1997; Northoff, 2002a,b); and modulation of the primary visual cortex by prefrontal cortical regions (Lamme, 2004).

Cognition-emotion interactions are crucially regulated via top-down modulation from the medial prefrontal cortex (MPFC) to the amygdala (Davidson, 2002; Pessoa et al., 2002; Pessoa and Ungerleider, 2004) and the insula (Nagai et al., 2004). The insula has a tense and reciprocal relation to other subcortical medial regions, for instance the hypothalamus and the periaqueductal gray (PAG; Panksepp, 1998a,b). Both the amygdala and the subcortical medial regions play an important role in the regulation of internal somatic functions, whereas the medial prefrontal cortical regions are predominantly associated with emotional processing (Phan et al., 2002; Murphy et al., 2003; Northoff and Bermpohl, 2004). All these three regions—the MPFC, the amygdala and subcortical medial regions—have tense and reciprocal connections. Therefore, circular modulation between all these regions may be assumed.

Possibly, there is not only a top-down modulation, but also a bottom-up modulation. A hierarchically lower region modulates the activity in a hierarchically higher region by means of the bottom-up modulation. In this way, subcortical midline regions may, for instance, modulate neuronal activity in the MPFC via the insula. Accordingly, it may be assumed that top-down/bottom-up modulation co-exists in the same region at the same time, which may, from a functional perspective, lead to reciprocal adaptation between emotional processing and processing of internal body functions. The simultaneous occurrence of top-down/bottom-up modulation corresponds to a psychological default-perspective wherein emotional awareness predominates over somatic awareness. The experience and awareness of internally or externally generated emotions comes to the fore, whereas the awareness of the body remains in the background. In this way, a predominating external focus may be explained, in which attention is directed to other persons and events in the outside world. In contrast, the internal focus—that is the attention to one’s own body—remains in the background.

The functional equilibrium between the top-down and bottom-up modulation may be disturbed in the course of regressive processes and defence mechanisms connected with these. *Regression* may be understood as a re-actualization of earlier functional levels and the concomitant dominance of somatic reactions instead of emotional and cognitive patterns of reaction. *Somatization* may be characterized as a specific form of mental processing and intensive perception of somatic disturbances. The mechanisms of somatization can be paradigmatically observed in depressive patients who often experience somatic symptoms—especially autonomous-vegetative symptoms—in a subjective way. It may be hypothesized that somatization in depression is correlated with a disturbed equilibrium of top-down/bottom-up modulation between emotional processing and processing of internal body signals.

At a functional level, somatization may point at the predominance of internal processing of the body in contrast to emotional processing. Primary signals of the internal controlling centers of the body are processed, whereas processing of the emotional stimuli originating either internally or externally remains in the background. The balance between internal processing of the body and emotional processing is adapted to a new functional level in the following course. Accordingly, it can be assumed that depressed patients with strong somatization react much more intensively to internal stimuli from the body than to internal or external emotional stimuli. Furthermore, depressed patients show extremely strong autonomous-vegetative reactions (for instance heart rate variability; Bär et al., 2004). Finally, depressed patients show reduced reactions to externally induced emotions, for instance in the context of social interaction. This was already underlined by results from earlier studies on communication in depression (see Coyne, 1976a,b, 1985; Coyne et al., 1987). From the psychological perspective, somatization is reflected by an increased awareness of one’s own body and the internal functions of the body. Depressed patients shift their attention away from their own or other’s emotions to their own body functions. Depressed patients do not observe their own emotions, but rather their own body functions. They do not observe others’ emotions, but rather their own body. At the neuropsychological level, this increased internal focus is reflected in changes in attention and theory of mind, which was shown empirically both in theory of mind tasks (Inoue et al., 2004) and in findings of attention deficits (Paradiso et al., 1997; Murphy et al., 1999, 2001; Sheppard and Teasdale, 2004). Recent neuroimaging studies (Paulus and Stein, 2010; for review Wiebking et al., 2010) support the notion that depressed patient show increased somatization related to altered brain functioning. Wiebking et al. (2010), for example, investigated the neural correlates of interoception in healthy and depressed subjects using the Body Perception Questionnaire (BPQ) and a well-established heartbeat perception task in fMRI. They found that depressed patients showed significantly higher scores in the BPQ and left anterior insula signal changes correlated with depression severity.

Furthermore, Ernst et al. (2013, 2014) investigated the neural correlates of interoception and its relationship to empathy. They found that preceding interoceptive awareness period significantly enhanced neural activity during empathy in bilateral anterior insula and various cortical midline regions. This suggests a close relationship between interoception and empathy; thereby, interoception seems to be implicated to yielding empathy.

### Reciprocal Modulation and Introjection

Neuroimaging studies showed a pattern of opposite signal changes in the medial and lateral prefrontal cortex (LPFC) during emotional-cognitive interaction (Goel and Dolan, 2003a,b; Northoff and Bermpohl, 2004; Northoff et al., 2004). These results correspond to the assumption of functional mechanisms of reciprocal modulation and reciprocal reduction during emotional-cognitive interaction. *Reciprocal modulation* is defined as signal changes in opposite directions (i.e., increases and decreases of signals). It is already known that emotional processing (when an emotional picture is perceived) leads to an increase of signals in the MPFC regions as well as to a decrease of signals in the LPFC (Phan et al., 2002; Murphy et al., 2003; Northoff et al., 2004).

In contrast, cognitive tasks (e.g., judgment or assessment) produce an opposite pattern of signal increases in the LPFC and signal decreases in the MPFC. This corresponds to the functional mechanism of reciprocal modulation (Northoff et al., 2004; Boeker and Northoff, 2018): the experience of the outside world is changed to an experience of the inner self (internal focus). Analogous patterns of reciprocal modulation were also observed in other cortical regions, for instance in the medial and lateral orbitofrontal cortex, in the right and left motor cortex, in the striatal and extra-striatal visual cortex, in the subgenual anterior cingulum and in the right prefrontal cortex, in the sub/pre- and supergenual anterior cingulum as well as in the visual and auditory cortex (overview in Northoff et al., 2004).

On the basis of the mentioned empirical results, it may be assumed that emotional-cognitive interaction is associated with the functional mechanism of reciprocal reduction: if a cognitive task comprises an emotional component (e.g., when assessing an emotional picture), fewer signal decreases result in the MPFC regions and simultaneously fewer signal increases in the LPFC regions. This process was characterized as *attenuation* (synonym: decrease; see Northoff et al., 2004). A reciprocal attenuation can be referred to because this process occurs in the medial as well as in the lateral prefrontal cortical regions in opposite directions (i.e., fewer signal decreases and increases).

It may be assumed that the reciprocal modulation and attenuation can be altered in introjective mechanisms at a neuropsychodynamic level.* Introjection* is—in an operational definition—characterized by the shift of object focus from the outside to the inside in subjective experience. The subject-object relationship is no longer directed to the outside, but to the inside. The experience of the outside is changed into an experience of the inner self.

The defence mechanism of introjection can be paradigmatically observed in depressed patients. These patients tend to internalize their conflicts with others and shift the aggression which was primarily directed towards others, against their own self. It may be hypothesized that this introjection in depression may be associated with the abnormal reciprocal modulation during emotional-cognitive interaction (Deci et al., 1994; Adroer, 1998; Malancharuvil, 2004).

From a functional and psychological perspective, introjective processes in depressed patients may be related to disturbances of emotional-cognitive re-adaptation. Depressed patients are no longer able to adequately assess their own emotional and body experience. The assessment of one’s own conditions is “subjectively” distorted and decoupled from “objective” reality. This subjective distortion is seen in a marked negativity of the assessment of one’s own emotions and the body, and also of the assessment of others’ emotions and the events in the outside world. This extreme negativity corresponds to the “negative or attentional bias” (Elliott et al., 1998, 2000, 2002; Gotlib et al., 2004). The results of a study on the perception of faces (Gotlib et al., 2004), for instance, pointed out that the “negative or attentional bias” may be connected to interpersonal dysfunction in depressed patients. Further studies are necessary to explain how and why the “negative or attentional bias” apparently induces a decoupling of the “subjective” assessment from the “objective” reality.

On the basis of the generated empirical results, it may be assumed that the disturbed reciprocal modulation and attenuation during emotional-cognitive interaction in depression is of outstanding importance for the development of introjective defence and compensation mechanisms. This notion has been supported by several neuroimaging studies (Mayberg et al., 1999; Brody et al., 2001; Drevets, 2001) which showed that depressive patients show both increased neural activity in the ventromedial prefrontal cortex (VMPFC) and decreased neural activity in the dorsolateral prefrontal cortex (DLPFC) as compared to healthy subjects. Thus, maladjustment of reciprocal modulation and attenuation might account for both emotional and cognitive deficits in depressive patients. Furthermore, Zimmermann et al. (2015), in their seminal psychotherapy study, applied multilevel mediation analyses; the results showed that post-treatment differences in interpersonal problems and introject affiliation were mediated by the higher number of sessions; and follow-up differences in depressive symptoms were mediated by the more pronounced application of psychoanalytic techniques. Furthermore, they also found some evidence for indirect treatment effects via psychoanalytic techniques on changes in introject affiliation during follow-up. They concluded that these effects provide support for the prediction that both a high dose and the application of psychoanalytic techniques facilitate therapeutic change in patients with major depression.

### Modulation Through Functional Unit and Sensori-Motor Regression

A further example of functional mechanisms of emotional-cognitive interaction is the development of functional units in different brain regions over time. Such transient functional units could be identified on the basis of psychophysiological features or functional connectivity in the involved regions (Friston, 1998, 2003; Friston et al., 1998a,b, 2003; Friston and Price, 2003). The cortical midline structures (CMS, Northoff and Bermpohl, 2004), for instance, show a continuously high level of neuronal activity also under resting state conditions (e.g., when passively regarding a fixation cross; Gusnard and Raichle, 2001; Gusnard et al., 2001; Mazoyer et al., 2001; Raichle, 2001; Raichle et al., 2001). Furthermore, the regions within the cortical midline structures are characterized by dense anatomical connections. In addition, investigations on the functional activity in the CMS found an increase in functional activity in the CMS between anterior and posterior CMS regions in the resting state, whereas this connectivity decreased during active cognitive tasks.

The participation of both anterior and posterior midline structures is in line with results from further studies on cognitive, emotional and social processing (overview in Northoff and Bermpohl, 2004). Furthermore, signal decreases were found both in the orbito-medial prefrontal cortex (OMPFC) and the parietal cortex (PC) during cognitive tasks requiring attention. In addition, these regions show an increased circular connectivity (Greicius et al., 2003, 2007; Raichle, 2003). In summary, these empirical results deliver convincing evidence for the existence of the CMS and a functional unit, which is particularly active and cohesive in the resting state (Greicius et al., 2003; Wicker et al., 2003a,b,c).

With this in mind, it may be assumed that modulation through functional unit is altered in regressive processes involving sensori- and motor functions. Sensori-motor regression can be defined as a defence and compensation mechanism, which is activated when conflicts and anxieties can no longer be solved by means of cognitive and emotional functions and when somatic and particularly sensori-motor functions are involved. The defence mechanism or the sensori-motor regression can be paradigmatically observed in patients with catatonia (“scared stiff”; Böker, 2000a,b, 2001; Böker et al., 2000a,b). It may be hypothesized that the sensori-motor regression in catatonia is connected with altered modulation by functional unit in the CMS (overview in Böker et al., 2000a,b; Northoff et al., 2003, 2004). This hypothesis has been investigated in an fMRI study in catatonic patients with an underlying affective or schizoaffective psychosis (Northoff et al., 2002). They found significantly altered activation patterns in orbitofrontal and premotor cortex during negative emotional stimulation which correlated significantly with affective, behavioral, and motor alterations in catatonia. It was concluded that orbitofrontal cortical dysfunction and related alterations in medial prefrontal and premotor cortical activity may account for lack of emotional control with consecutive sensori-motor regression as an “immobilization by anxieties” in catatonia (both in depression and schizophrenia), where regression to somatic defense mechanisms is paradigmatically observed.

### Self-Referential Processing, Ego-Inhibition and Decoupling of the Self

Certain sensory stimuli are related to one’s own person, contrary to other stimuli which are rather related to other persons and the outside world. Accordingly, self-referential stimuli can be differentiated from non-self-referential stimuli (Northoff and Bermpohl, 2004; Northoff and Boeker, 2006). This differentiation is valid not only for sensory stimuli, but also for emotional and cognitive stimuli. From a functional perspective, self-referential processing points at a simple distinguishing process, the distinction between self and non-self. This process is decisive for the distinction between one’s own stimuli and those from others, and therefore for the distinction between self and the outside world. Moreover, self-referential processing may be a pre-requisite condition for the development of a concept of one’s own self, the so-called mental or phenomenal self as a subject of experience (Northoff et al., 1997, 2000, 2005; Panksepp, 1998a,b; Damasio, 1999; Northoff, 2004).

From a psychological perspective, self-referential processing may be manifest in the possibility of a subjective experience of one’s own self or ego (both terms are used synonymously in the following; see Freud, 1914; Kohut, 1977; Dennecker, 1989). By marking certain stimuli as self-referential, they can be experienced subjectively, i.e., from the individual subject’s or ego perspective (Northoff and Boeker, 2006). Owing to the fact that, besides the internal and sensory stimuli of one’s own body, emotions and cognitions are also examined with regard to their self-reference, emotions and cognitions can be attributed to one’s own self. The subject constituted primarily by one’s own body is “filled”, so to speak, with certain related emotions and cognitions.

The development of neuropsychodynamic hypotheses of altered self-reference in depression is based on the investigation of the emotional-cognitive interaction in depressed patients, which focussed on the neurophysiological correlates of depressive inhibition and the neurophysiological substrates of negative cognitive schemes and the neuropsychological deficits (overview in Northoff, 2002a,b; Northoff et al., 2005, 2007; Böker and Northoff, 2005, 2010; Grimm et al., 2006, 2008, 2009; Walter et al., 2009; Boeker et al., 2012). In addition to the emotional-cognitive interaction, semantic representations (and their neural correlates) play an important role in depression. Beyond the concept of cognitive control, psychodynamic (and neuropsychodynamic) models of depression focus on the development of the individuals’ capacity to regulate their emotional states in significant interactive relationships during childhood and through the re-construction of the representation of the self, of significant others, and relationships (see Messina et al., 2015, 2016a,b). Furthermore, consistent with psychodynamic (and neuropsychodynamic) models of depression, psychological functions associated with the default mode system include self-related processing, semantic processes, and implicit forms of emotion regulation (Messina et al., 2016a).

Rumination is a form of self-referential processing, which is the process of relating information to the self. In a meta-analysis of neuroimaging studies focused on self-referential processing, Northoff et al. (2006) found that commonly activated regions lie in dorsal and ventral areas of the medial prefrontal and anterior cingulate cortices, as well as the posterior cingulate cortex and precuneus. These regions have been termed cortical midline structures (Northoff and Bermpohl, 2004) and somewhat overlap with the intrinsic default mode network (DMN; Raichle et al., 2001). A recent review (Nejad et al., 2013) of neuroimaging studies on rumination, self-related processing, and depression found that the anterior cortical midline structures play an important role in maladaptive rumination in major depression.

To sum up, depression may be characterized by reduced neuronal activity in the left DLPFC and increased activity in the right DLPFC. The neuronal activity in the left DLPFC cannot be modified by emotional valence. The severity of depression correlates with the activity in the right DLPFC. Connected with the reduced deactivation in the pregenual ACC (DMN), depressed persons cannot shift their attention from themselves to the outside world (Grimm et al., 2008). The degree of helplessness and the severity of depressive symptoms correlate with the reduced deactivation in the perigenual anterior cingulate cortex (PACC) and posterior cingulate cortex (PCC). The signal intensities in different subcortical and cortical midline regions (DMPFC, supragenual anterior cingulate cortex (SACC), precuneus, ventral striatum (VS), DMT) were reduced significantly. On the basis of these empirical results, it may be concluded that the increased negative self-attributions—as typical characteristics of an increased self-focus in depression—may result from altered neuronal activity in subcortical-cortical midline structures in the brain^1^ (especially from hyperactivity in the cortical-subcortical midline regions and hypoactivity in the lateral regions). On the basis of neuropsychological, neurophysiological and neurochemical findings and, as we mentioned, psychodynamic dimensions of depression, neuropsychodynamic hypotheses on the disturbed self-reference in depression were developed and related to psychodynamic and specific neuronal mechanisms of depression.

Finally, the decoupling of the self characterizes the extreme form of the altered self-related processes in depression, including complete loss of self. A typical example of decoupled self experience may be encountered in severely depressed patients with psychotic symptoms, e.g., nihilistic delusion (Cotard Syndrome).

## Dysbalance of Resting State Activity in DMN and Mental Reactivation of Early Object Loss

In the neuropsychodynamic framework presented here, it may be hypothesized that altered resting state activity^2^ in depression is a pre-disposition for reactivation of early object loss experiences in the subject. There is ample evidence for resting state abnormalities in depression. For example, PET-studies in major depression found decreased resting state activity, especially in the lateral anterior cortical midline regions (PACC, VMPFC, compare Mayberg et al., 1999; Mayberg, 2002, 2003a,b; Phillips et al., 2003). Alterations in neural activity were shown in ventral regions of the so-called DMN in depressed patients (reduced deactivation, that is negative BOLD-reactions, see Greicius et al., 2007; Grimm et al., 2009; Sheline et al., 2009). Furthermore, a translational meta-analysis of resting state studies in depressed patients and in animal models confirm resting state *hyperactivity* in ventral, cortical midline regions (PACC, VMPFC; Drevets and Raichle, 1998; Drevets, 2000, 2001; Fitzgerald et al., 2007; Drevets et al., 2008; Alcaro et al., 2010; Price and Drevets, 2010). In contrast to the anterior midline regions, posterior midline regions (PCC, precuneus/cuneus) and the superior temporal gyrus (STG) show *hypoactivity* in the resting state (Heinzel et al., 2009; Alcaro et al., 2010). The hyperactivity in the anterior midline regions and the hypoactivity in the posterior midline regions result from a disturbed balance between anterior and posterior midline regions in acute depression and a disturbance in the DMN in depression (Raichle et al., 2001; Buckner et al., 2008).

How can these results be related to psychodynamic mechanisms, especially the reactivation of early loss in childhood? Early traumatization causes alterations in developing processes which may contribute to the development of early immature defence mechanisms (Feinberg, 2011). Traumatization in early childhood was found in a large subgroup of depressed patients (experiences of loss, divorce of parents, physical or sexual abuse; see Böker, 2000a,b; Nemeroff et al., 2003; Gabbard, 2005, 2014). Traumatic life-events may cause biological alterations on the genetic, hormonal or anatomic-structural level (Feder et al., 2009). It may be hypothesized that traumatic life experiences interfere with the development of the VMPC and especially the ventral anterior subcortical-cortical midline regions as an essential part of the DMN. Anterior midline regions are especially involved in the processing of the degree of self-reference of different stimuli, whereas the posterior regions are likely to be involved in the processing of social and non-self-related stimuli (Qin and Northoff, 2011).

Furthermore, it may be assumed that early traumatic experience of object loss is associated with the desperate attempt to relate the self to the lost object in order to develop a self-object relationship and to experience the lost object as a self-object. In connection with this, possible hyperactivation, especially in the anterior midline regions, may be induced, which finally contributes to a disbalance in the posterior midline regions.

The retrieval of early traumatic experiences in the context of current experiences of object loss triggers a reactivation of the same neuronal patterns used in the early development of a relationship to the object with a consecutive hyperactivity in the anterior midline regions and a disbalance in the posterior regions and in the DMN.

**Figure 1:**
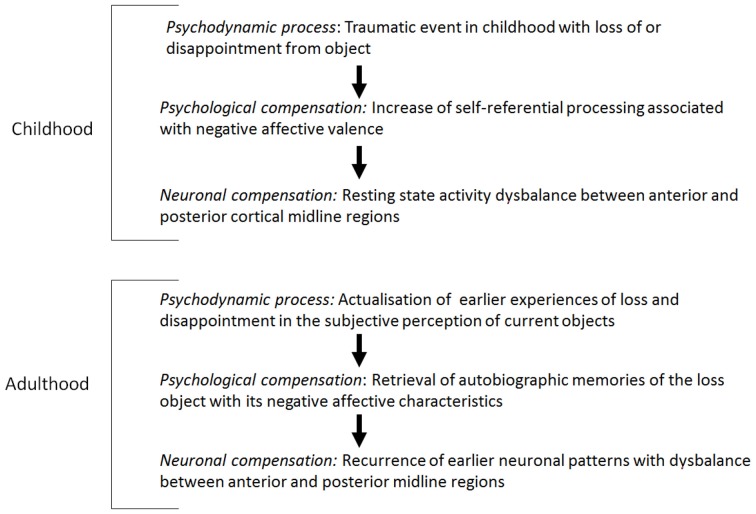
Resting state activity: dysbalance and mental reactivation of lost objects.

In view of the early object loss, depressed patients develop a psychological pre-disposition to attribute a high degree of self-reference to lost or disappointing objects. Therefore, a neuronal pre-disposition for the development of hyperactivity in the resting state in anterior midline regions, when object loss is experienced in adulthood, is induced. Accordingly, it can be hypothesized that the psychological and neuronal predisposition correspond with the psychodynamic predisposition, the reactivation of early object loss. When actual stimuli induce less stimulus-induced activity in the anterior midline regions because of the abnormally increased resting state activity, the brain keeps predominantly occupied with itself, i.e., that the earlier experienced contents and the objects are reactivated by the increased resting state activity accordingly or that the representations of the earlier objects are represented in the actual context (see Figure 1; see Boeker and Northoff, 2018).

### Reduced Resting State-Stimulus-Interaction and Introjection in Correlation With Negative Emotions

Resting state-stimulus-interaction includes the interaction between two different modes of neural activity: the intrinsic activity of the brain and the stimulus-induced activity (induced by stimuli from the outside world and/or the own body). It may be hypothesized that resting state-stimulus-interaction in depression is reduced because of increased resting state activity and that the resting state-stimulus-interaction is associated with introjective processes in the interaction between self and the object world connected with negative emotions. Functional activation paradigms (emotional, cognitive) showed dysfunctional activation patterns, especially hyperactivity, in the ventral cortical midline regions in patients with major depression during resting state and emotional stimulation (Elliott et al., 1998, 2002; Mayberg et al., 1999; Davidson, 2002; Mayberg, 2002, 2003a,b; Davidson et al., 2003; Canli et al., 2004; Fu et al., 2004). In other brain regions, especially in the reward system (VS/n.accumbens, right and left amygdala), dysfunctional activation patterns were found during positive and/or negative emotional stimulation in major depressive disorder (MDD; Kumari et al., 2003; Canli et al., 2004; Lawrence et al., 2004; Surguladze et al., 2004). These dysfunctional activation patterns may be interpreted as the neurophysiological basis of the “negative affective bias,” that is the focussing of negative emotions which is related to the inability to process positive emotions (Mayberg, 2003a,b; Phillips et al., 2003; Grimm et al., 2009; Heinzel et al., 2009; Heller et al., 2009). The reduced deactivation in cortical-subcortical regions was correlated with the severity of depression and the degree of hopelessness. A direct association between reduced resting state-stimulus-interaction and the severity of depression may be assumed (Grimm et al., 2009).

Further studies focussed on the biochemical basis of the reduced resting state stimulus interaction. It could be shown that increased resting state activity in the PACC is associated with the concentration of the neurotransmitter glutamate (Zarate et al., 2006; Maeng and Zarate, 2007; Northoff et al., 2007; Maeng et al., 2008; Walter et al., 2009; Alcaro et al., 2010; Sanacora, 2010). A disbalance between neuronal inhibition and excitation in the anterior midline regions in depressed patients may be assumed.

The induced resting state activity in the brain inhibits the neuronal processing of stimuli from the outside world. Stimuli from the outside world cannot be related to one’s own self or connected with emotional valence. Nevertheless, these psychological mechanisms are still active and are mediated by the induced resting state activity. From a psycho-energetic perspective, the energy which is usually used for the development of self-relatedness and the connection with emotions (emotional valence) is related to early stimuli which are related to early object loss and the induced resting state activity.

The change from current to early stimuli may contribute to an increase of introjective processes in a neuropsychodynamic perspective. The reactivated stimuli from the past are related to the self and negative emotional valence. Stimuli which are connected with early object loss are introjected and related with negative emotions. It may be assumed that this process corresponds with an introjective type of depression (Blatt, 1998) which is characterized by an increased interpersonal relatedness.

### Reduced Stimulus-Resting State-Interaction and the Loss of Current Object Relations

**Figure 2:**
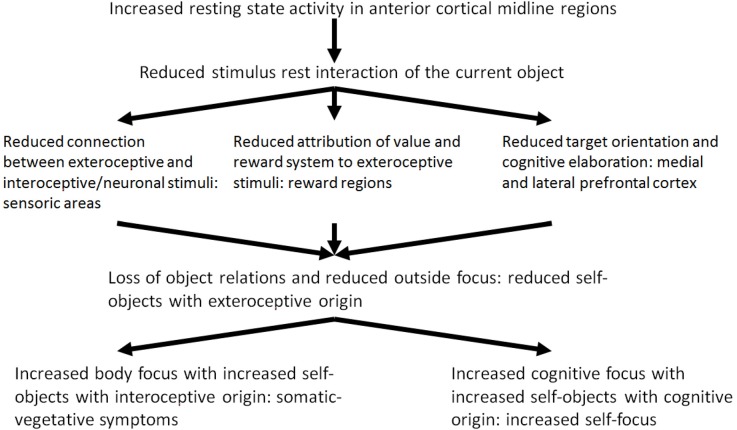
Reduced stimulus-rest-interaction, reduced outside focus and increased self-focus.

It may be hypothesized that the modulation of the resting state activity by means of stimulus-induced activity and the stimulus resting state interaction in depression is reduced because of the increased resting state activity. Reduced stimulus resting state interaction probably leads to dysfunctional development of the neuronal structure and organization which is associated with dysfunctional processing of current experiences of loss. The reduced stimulus resting state interaction can be seen in three different patterns: (1) interoceptive stimuli no longer modify the resting state or baseline activity of the brain (Wiebking et al., 2010); (2) exteroceptive stimuli are no longer associated with value and reward (Kumar et al., 2008; Dichter et al., 2009; Pizzagalli et al., 2009; Smoski et al., 2009; Sajonz et al., 2010); and (3) exteroceptive stimuli no longer induce or constitute cognitive processing (Goel and Dolan, 2003a,b; Northoff et al., 2004; Grimm et al., 2006; see Figure 2, see Boeker and Northoff, 2018).

On the basis of these empirical results, Phillips et al. (2003) and Mayberg (2002, 2003a) developed a model of the altered reciprocal functional interaction between ventromedial and DLPFC in MDD (model of ventro-dorsal dissociation) where reciprocal modulation of neural activity in depression is reduced because of reduced deactivation in the medial regions and reduced activation of the DLPFC (Carhart-Harris et al., 2008; Grimm et al., 2008). The disturbed modulation of the lateral prefrontal cortical resting state most probably contributes to a reduced stimulus-induced activity triggered by cognitive stimuli. From a neuropsychodynamic perspective, the reduced stimulus resting state activity and the reduced exteroceptive neuronal interaction contributes to a reduced constitution of objects which also results from the reduced emotional valence and reward. Current object relationship experiences lose their emotional significance. Reduced reciprocal modulation—as the third part of the reduced stimulus resting state interaction—contributes to a reduced activation of cognitive processes by exteroceptive stimuli. In this way, the “object cathexis” is reduced (Carhart-Harris et al., 2008). Furthermore, it may be assumed that the disturbed reciprocal modulation may be an adaptive mechanism by which the depressive self is finally disconnected from the significance of current object relationship experiences. From a psycho-energetic perspective, the result is a further reduction of object cathexis. The depressed patient attempts to constitute and cathect compensational objects from his inner world. The inner world encompasses interoceptive stimuli of the body and cognitive stimuli instead of external objects.

### Reduced Interaction of Exteroceptive Stimuli

Wiebking et al. (2010) studied the neuronal activity during exteroceptive and interoceptive perception (heartbeat and counting of the cardiac frequency) in relation to the resting state activity of the brain. Exteroceptive stimuli (heartbeat) contributed to a neuronal activation in the bilateral anterior insula in depressed patients, if it was related to the previous resting state activity of the brain. But if the exteroceptive-induced activity was viewed independently from the previous resting state activity, significant differences were found between healthy persons and depressed patients. The resting state activity in depressed was increased compared to healthy persons, as the reduced deactivation during stimulus-induced activity had shown already. Thus, the increased resting state activity in the bilateral anterior insula contributed to a reduced interaction of the exteroceptive stimulus with the previously increased resting state activity. Analogous results were not found during the interoceptive stimuli (counting of the heartbeat), by means of which the specificity of the reduced stimulus-resting state-interaction during exteroceptive stimuli was underlined.

The importance of the increased resting state activity in the bilateral anterior insula was further supported through its correlation with the severity of depression: the higher the resting state activity in these regions, the more severe the depression was experienced in the self-assessment beck depression inventory (BDI). Furthermore, depressed patients showed increased scores for body perception and stress (BPQ). This result is consistent with the increased perception and attention to one’s own body and the unspecific somatic symptoms, which are characteristic of depression. Whereas the BPQ-scores in healthy persons correlated with the resting state activity in the insula significantly, this correlation was not found in depressed patients: therefore, it could be concluded that the increased resting state activity in depressed was decoupled from the perception and experience of one’s own body. The increased resting state activity and the consecutively reduced stimulus-rest-interaction are psychologically and psychopathologically of great importance.

The second component of the reduced stimulus-rest-interaction relates to the processing of value and reward. The reward network includes central regions the ventral tegmental area (VTA), the VS, and the VMPFC. Actual studies underline a reduced activity during the processing of reward tasks through exteroceptive stimuli just in these regions of depressed patients (Kumar et al., 2008; Dichter et al., 2009; Pizzagalli et al., 2009; Smoski et al., 2009).

### Reduced Stimulus-Rest-Interaction and Cognitive Processing

The third component of the reduced stimulus-rest-interaction relates to the reciprocal pattern of neuronal activity between the medial and LPFC which could be shown in different studies (Goel and Dolan, 2003b; Northoff et al., 2004; Grimm et al., 2006) and which were described as reciprocal modulation (Northoff et al., 2004). In healthy persons, exteroceptive stimuli lead to a deactivation, i.e., to negative BOLD-answers, in the anterior medial cortical regions, whereas they lead to an increased neuronal activity in the lateral prefrontal cortical regions (e.g., in the DLPFC) during cognitive and emotional stimulation. Contrary, in depressed patients—as already mentioned—not only a hyperactivity in the ventral cortical midline regions was found, but also a hypoactivity in the left DLPFC as well during emotional as during cognitive processing either (Davidson et al., 2003; Lawrence et al., 2004; Keedwell et al., 2005). On the basis of these results, Phillips et al. (2003) and Mayberg (2003b) proposed a model of altered reciprocal functional connections between ventromedial and DLPFC in major depression (model of the ventro-dorsal dissociation in major depression). The reciprocal modulation is reduced in depression because of the decreased deactivation in the medial regions and the decreased activation of the DLPFC (Grimm et al., 2008; Carhart-Harris and Friston, 2010), i.e., there is an opposite activity in the medial and LPFC. When the medial regions are strongly deactivated, a strong activation in the lateral regions results, when the lateral regions are less activated, a less deactivation results in the medial regions. Therefore, exteroceptive stimuli do not lead to a decreased deactivation in the medial cortical regions, but also to a decreased activation in the lateral regions (DLPFC), which are especially associated with cognitive processing (Grimm et al., 2006, 2008, 2009). The disturbed modulation of the lateral prefrontal cortical rest activity probably leads to a decreased stimulus-induced activity, which is triggered by cognitive stimuli.

Of what significance are these three different components of the reduced stimulus-rest-interaction in a psychodynamic context? In depression, exteroceptive stimuli do not induce the existing resting state activity of the brain any longer. The reduced stimulus-resting state activity contributes to a reduced exteroceptive-neuronal interaction. It may be assumed accordingly, that the exteroceptive stimuli and the objects with are related to them are introjected with less probability. The consecutive reduced constituting of the objects also results from the second component of the reduced stimulus-rest-interaction, the reduced processing of value and reward. The neuronal activity of the reward system—as the basis of processing of value induced by stimuli—is extremely reduced in depression. In a psychodynamic context it can be assumed that the selection of objects and the possibility to identify with them is more and more reduced because of the reduced ability of processing of value of stimuli. Actual object relation experiences get increasingly unimportant.

The reduced reciprocal modulation—as third component of the reduced stimulus-rest-interaction—implies that exteroceptive stimuli no longer induce the activation of cognitive processes (e.g., goal-oriented cognitions). This interferes with the “*object cathexis*,” which Carhart-Harris and Friston (2010) associate in a neuropsychological perspective with goal-oriented cognition and activity within the DLPFC. The important thing here is that the reciprocal modulation itself is not disturbed in depression, but rather the causes of the reduced medial deactivation have to be explored. These may consist in the reduced rest activity and the reduction of exteroceptive stimuli, in which the latter is connected with the reduction of the previous exteroceptive-neuronal processing of values. Neuronal activity is not induced in the medial cortical regions by the incoming exteroceptive stimuli. In this perspective, the apparent abnormal reciprocal modulation in depression represents a process of compensation aiming at adapting the brain with regard to the processing of exteroceptive stimuli. Accordingly, the neurophysiologic problem in depression is not a lesion or a deficit of the reciprocal modulation, but the maintenance and the adaptive function of this mechanism. The depressive self is uncoupled from the experienced significance of actual object relation experiences finally. In a psych energetic perspective, a reduction of the object cathexis results.

Which are the mechanisms of compensation which are developed by the depressed patient in this situation? To answer this question Freud’s metaphor of an open wound may be picked up: The depressed tries to constitute compensatory objects and to cathect them. In view of the inability to constitute objects of exteroceptive origin, i.e., from the outside, and to introject them, the depressed patient constitutes and introjects objects from the inside. The internal environment includes interoceptive stimuli from the body and cognitive stimuli. Instead of exteroceptive-interoceptive and exteroceptive-cognitive connections, neuronal-interoceptive and interoceptive-cognitive connections are developed from now on. By this, the depressed attempts to compensate the loss of exteroceptive stimuli. In a psychodynamic perspective external objects, i.e., objects of exteroceptive origin from the outside, are replaced by internal objects, i.e., objects of interoceptive and cognitive origin. The outstanding aim is to constitute objects and to develop object relations. Therefore, in a self-psychological perspective, this can be understood as a compensation of the loss of external or sensory self objects by internal or somatic and cognitive self objects.

Regarding psychopathological symptoms, the constitution of internal or somatic and cognitive self objects contributes to the development of somatic symptoms. The body is perceived in an altered way (see Wiebking et al., 2010). If the constitution of cognitive self objects dominates, the depressed suffers from distinctly distorted, negative cognitive schemes and ruminations, which are experienced as painful and tormenting. Because of the loss of exteroceptive objects the focus of attention is directed to one’s own self and the self objects of cognitive origin. In a phenomenological perspective this was described by us as increased cognitive processing of one’s own self with ruminations and a disbalance between the experiential and the analytical self-focus.

## Neuropsychodynamic Approach to Treatment of Depression

How can we use the neuropsychodyamic model of depression for psychotherapeutic interventions in the treatment of depressed patients? Due to the multidimensionality and heterogeneity of depressive disorders, it is of essential importance to adapt the applicable somatotherapeutic and psychotherapeutic interventions to the specific conditions of each single case. The concept of depression as psychosomatosis of emotion regulation and the neuropsychodynamic model of depression may promote this individualized procedure (see Böker, 2017). The main aim of the psychotherapy of depression is to overcome the defensive strategies of the depressed patients and their intrapsychic, interpersonal, and psychosomatic vicious circles (Mentzos, 1995; Böker, 2002, 2006, 2009, 2017). Further therapeutic consequences of the neuropsychodynamic approach to depression involve the necessary emotional attunement in psychoanalytic psychotherapy of depressed patients and the adequate timing of therapeutic interventions (see Stern, 1983, 1985; Böker, 2003a,b; Boeker et al., 2013; see Table 1).

**Table 1:** Neuropsychodynamic approach, psychotherapeutic attitude and therapeutic focus.

**Depression**
- Dysfunction of cortical midline structures (CMS) - Arousal (Hyperactivity CMS) - Disturbed self-referential processing - Intensified processing of the body
**Therapy**:
- Acute and severe depression: state variables - Longer course: trait variables
**Neuropsychodynamic psychotherapy**:
- Phase typical, stepwise adapted focus - Beginning: - Containment - Being aware of anxiety, agitation and cognitive dysfunction - Course: - Increasing focus on conflictuous self-worth regulation and relationship expectations - Long-time course (recidivism, double depression, loss of psychosocial functions, chronicity): psychotherapeutic maintenance strategies

A stepwise adapted therapeutic focus, which takes state-variables of depression as well as trait-variables into account, is necessary. Particularly with regard to the actual emotional and cognitive state (see Böker and Grimm, 2012) and to the recidivism and chronification in the long-time course either, the importance of the hyperarousal of depressed patients, as subjective and clinical expression of the hyperactivity of the cortical midline structures, which was pointed out in this contribution, has to be kept in mind. Agitation and anxiety of the depressed patient require an appropriate containment, especially in the beginning of the treatment. In the long-time treatment course structural aspects of the personality of the patients move more and more into the center (e.g., dependency in relationships), furthermore the effects of experienced traumatization in another subgroup. The synergistic effects of pharmaco- and psychotherapy (as an expression of top-down- and bottom-up-mechanisms) are often the starting point for a combination therapy.

Psychoanalytic psychotherapy focusses primarily on the conflictuous forms of self-worth regulation and relationship expectations, especially in the long-time course. Generally, due to network dynamics and gene-expression, a sufficiently long duration of the treatment is necessary, and “psychotherapeutic maintenance strategies” are often necessary (Schauenburg and Clarkin, 2003).

The adequate timing of therapeutic interventions (in the sense of “now moments”, see Stern, 1983) is especially important in the treatment of depressed patients. In this context, a specific problem of treatment technique derives from the encounter with different levels of symbolization and a possibly discontinuous process of de- and re-symbolization, which may be connected with a temporal decoupling of the psychic process and a reoccurrence of manifest depressive symptoms (see Böker, 2003a,b; Böker and Grimm, 2012; Böker, 2017). This discontinuity may also contribute to difficult countertransference feelings (e.g., guilt, shame, aggression, doubtfulness, self-criticism), similar to those feelings the depressed patient is suffering from. Furthermore, this may hinder the psychotherapist in his/her necessarily supportive and confident attitude. The insight into the neuronal and neuropsychodynamic mechanisms of depression (e.g., resting state hyperactivity, reduced rest-stimulus interaction) may help the psychotherapist to get along with this specific challenge in the therapeutic relationship and to use the countertransference feelings for therapeutic interventions in the further course.

Emotional experience evolves during the course of a psychoanalytic psychotherapy of depressed patients in the sense of a re-symbolization from the participation in a sensomotor affect through a trans modal change to an experienced emotion (“semiotic progression,” see Böhme-Bloem, 1999) by discovering the otherness of the other and being able to mourn the separation: “The capacity to experience loss and the wish to recreate the object within oneself gives the individual the unconscious freedom in the use of symbols” (Segal, 1957, p. 394). Segal concluded: “The process of symbol formation is, …, a continuous process of bringing together and integrating the internal with the external, the subject with the object, and the earlier experiences with the later ones” (p. 397).

The hypotheses which have been developed in the context of the neuropsychodynamic model of depression may be used for more specific psychotherapeutic interventions, aiming at specific mechanisms of compensation and defence, which are related to the increased resting state activity and the disturbed resting state-stimulus-interaction. Moreover, in a future “brain-based psychodynamic psychotherapy” of depression, the enabled processes of development and separation will be based on new experiences in the context of the therapeutic relationship.

Finally, the neurobiological effects of psychotherapy, though one of the most interesting and important challenges of a multidimensional psychotherapy research (for a methodological discussion, see Boeker et al., 2013), was not in the focus of the article. Because of the importance of this issue, we shortly mention recent neurobiological literature concerning neural effects of psychotherapy. A recent meta-analysis on neuroimaging findings of neural change in brain networks associated to emotion regulation after psychotherapy of depression by Messina et al. (2013) detected consistent changes in the DMPFC and in the posterior cingulated gyrus/precuneus, and in several areas in the temporal lobes in depression. A further meta-analysis by Kalsi et al. (2017) compared the effects of psychotherapy compared to antidepressant therapy on brain activity in depression. They found that patients undergoing psychotherapy showed an increase in the right paracingulate activity while pharmacological treatment led to a decrease of activation of this area. They interpreted this finding as supporting the hypothesis that psychotherapy increases top-down emotional regulation through self-knowledge and meaning processing. In a recent neuroimaging study in depressed patients undergoing psychodynamic psychotherapy, Buchheim et al. (2012) recently showed that psychodynamic psychotherapy in depressed patients reduces activation in the left anterior hippocampus/amygdala, subgenual cingulate and MPFC after 15 months.

## Conclusions and Outlook

A neuropsychodynamic approach to depression may be summarized as follows: the self and the changes in self-experience are core dimensions in depression and of psychoanalytical theories of depression. The experience of self-related depression can be characterized as the experience of the loss of the self. A mechanism-based approach was developed, focussing on the psychodynamic, psychological and neuronal mechanisms in healthy and depressed persons. On the basis of empirical results concerning emotional-cognitive interaction in depression, neuropsychodynamic hypotheses of the self in depression were developed.

In particular, it may be assumed that the empirically validated *increased resting state activity* in depression is a pre-disposition for the reactivation of experiences of early loss. The term “experiences of object loss” focuses not only on traumatic relationship experiences, but also encompasses the loss of the self in a significant relationship structure. Further, it may be hypothesized that the *resting state-stimulus-interaction* in depression is reduced because of the increased resting state activity and that it corresponds with introjective processes of the self in the relationship with objects (correlated with negative emotions).

The increased resting state activity in depression is especially associated with an increased resting state activity in the DMN. By means of this, changes in the complete spatial temporal structure of the intrinsic activity of the brain and the disbalance between DMN and executive network (EN) are induced. The reciprocal or negative interaction between DMN and EN is shifted in the direction of the DMN. This disbalance causes an abnormal increase in the internal mental contents, whereas externally oriented actions are decreased. The increased inward focus (with strong ruminations) and a reduced outward focus (with a reduced relationship to the outside world) are core symptoms in depression. The depressed patient is no longer able to differentiate between external stimuli and his own self (caused by the increased resting state activity in the DMN which cannot be modified by external stimuli).

The question of why adaptive mechanisms are activated in the disturbed context of the increased resting state activity may be answered by mentioning the central aim of these neuropsychodynamic mechanisms: to maintain at all costs the subjective existence of the self in view of the experienced threat of loss of the self. It is neither lesions nor disturbances of adaptive neuronal mechanisms which generate depressive symptoms, but rather increasingly dysfunctional mechanisms of compensation on the basis of the increased resting state activity.

As has been shown, different kinds of cognitive symptoms in depression may be connected to different neuropsychological functions, which in turn can be attributed to different forms of processing in the prefrontal cortex. These different forms of processing in the prefrontal cortex can be characterized by spatiotemporal patterns of neuronal activity, which are altered in a specific way during depression. In depression, there is hypofunction in the medial orbitofrontal cortex with consecutive changes in feedforward, feedback and re-entrant processing in the prefrontal cortex, anterior cingulate, DLPFC and VLPFC, respectively. The changes in prefrontal processing in depression are mostly related to an abnormal increase and synchronization in feedforward processing from the VMPFC, as well as to the resulting abnormal reduction of feedback and re-entrant processing.

Psychotherapeutic interventions in depression should focus on restoring these various forms of processing in the prefrontal cortex. In this context, emotional, cognitional and motor imagination, as well as working memory training, chronometrically oriented cognitive therapy and awareness of the time dimension play an important role in the psychotherapy of depression. These various inputs, based on neurophysiological mechanisms, could well complement existing psychotherapeutic approaches such as Psychodynamic Psychotherapy, Cognitive Behavioral Therapy (CBT), Interpersonal Therapy (IPT), Mindfulness-Based Cognitive Therapy (MBCT) and Cognitive Behavioral Analysis of Psychotherapy (CBASP). Considering the cognitive phenomenology described above and the underlying physiological mechanisms, new psychotherapeutic approaches may be developed in the future on the basis of the specific change in processing in the prefrontal cortex, in the sense of “phenomenologically and physiologically based neuropsychodynamic psychotherapy”.

The role of attribution, i.e., the subjective interpretation of events, has been widely studied within the context of depression (for a recent review see Rubenstein et al., 2016). However, neuropsychodynamic psychiatry (see Boeker and Northoff, 2018) encompasses a novel approach to psychopathology and its psychodynamic dimensions. Neuropsychodynamic psychiatry aims to complement and extend phenomenological psychopathology beyond the phenomenal boundaries of experience and thus towards the brain. Methodologically, this requires two-fold access: the neuropsychodynamic psychiatrist and psychoanalyst needs access to subjects’ experience while, at the same time, she/he requires access to the brain’s spontaneous activity. Furthermore, the knowledge about the neuropsychodynamic mechanisms of depression may support the psychotherapist to recognize and use her/his countertransference feelings for therapeutic interventions. In the neuropsychodynamic approach to depression, therapeutic relationship (empathy, countertransference, therapeutic alliance) is of outstanding importance. In fact, one of the authors, HB, underlined the importance of the therapeutic relationship in the psychotherapy of depressed patients in a recent publication (Boeker, 2017). We hope that the neuropsychodynamic approach may assist the therapist/psychoanalyst perceiving, considering, and understanding some of the specific characteristics of the “affect communication” between the depressed patient and the therapist, e.g., increased self-focus, increased body-focus, agitation in the context of resting state dysfunctions. Therefore, we underlined the importance of the emotional attunement in psychoanalytic psychotherapy of depressed patients and the adequate timing of therapeutic interventions (see “Neuropsychodynamic Approach to Treatment of Depression” section). The knowledge on neurobiological/neuropsychodynamic mechanisms (e.g., increased resting state activity) may help the psychotherapist to use her/his countertransference and to enable an appropriate containmemt (especially in the beginning of the therapy) and to adapt the therapeutical interventions in the long-time treatment course considering structural aspects of the personality of the depressed patient. This is strongly supported by recent evidence from therapeutic outcome studies in major depression (Blatt et al., 2010; Zimmermann et al., 2015) which showed that therapeutic relationship facilitates changes in negative self-representation (“introjective pole of depressive psychopathology”) and structural organization of patients’ inter-personal schemas (“anaclitic pole of depressive psychopathology”), leading to sustained therapeutic change.

Furthermore, semantic representations of the relationships represent an important inter-individual process in the therapeutic relationship and in the development of the patients’ capacities to regulate their emotional states through the construction of the self, others and relationships. On this way the therapeutic relationship is “a key tool for revisiting impaired or distorted representations of the self and relational objects” (Messina et al., 2016a). Some limitations of this neuropsychodynamic approach to depression concern the problem of investigating psychodynamic dimensions of depression by means of operationalized studies (see Böker and Northoff, 2010; Boeker et al., 2013). Further studies are necessary to validate the increased resting state activity in depression.

## Author Contributions

HB: main author, empirical studies on depression, development of hypotheses and concepts. RK: co-author, neuroscientific research, critical discussion of literature and concepts.

## Conflict of Interest Statement

The authors declare that the research was conducted in the absence of any commercial or financial relationships that could be construed as a potential conflict of interest. The reviewer MB and handling Editor declared their shared affiliation.

## Footnotes

^1^We do not understand the cortical midline areas as an unitary neural system but as an important component of neural integration which characterizes the coordination and modulation of neuronal activity over multiple brain regions. We desribed functional and effective connectivity and four principles of neural integration in the third section of the article (see Fox et al., 2005).

^2^Resting state activity in the brain represents the intrinsic activity of the brain and should be differentiated from the brain’s activity induced by somatic stimuli or outside stimuli.
